# Prevalence and Risk Factors for Absconding from an Open-Door, No-Restraint Inpatient Psychiatric Unit: A Single-Center Study in Italy

**DOI:** 10.3390/bs13010058

**Published:** 2023-01-08

**Authors:** Giovanni Napoli, Marcella Cannone, Marco Garzitto, Marco Colizzi, Matteo Balestrieri

**Affiliations:** 1 General Hospital Psychiatric Unit (GHPU), Department of Mental Health, Friuli Centrale Health University Authority, 33100 Udine, Italy; 2 Unit of Psychiatry, Department of Medicine (DAME), University of Udine, 33100 Udine, Italy; 3 Department of Psychosis Studies, Institute of Psychiatry, Psychology and Neuroscience, King’s College London, London SE5 8AF, UK

**Keywords:** mental health care, treatment, care settings

## Abstract

Absconding from inpatient psychiatric services has been associated with poor outcomes, putting the patient and community at risk and prolonging the recovery process. A retrospective study investigated the absconding rates and risk factors among patients admitted to an open-door, no-restraint inpatient psychiatric unit. Overall, the absconding rate was 4.5%, and the relative risk of absconding was higher for male, younger, and non-Caucasian patients as well as for those who had already absconded, were unknown to health services, compulsorily admitted, admitted for substance abuse, and in the first days of hospitalization. The findings of this study may have important public health implications.

## 1. Introduction

Estimated to occur at a rate of between 0.2% and 54.4% [1], absconding from inpatient psychiatric services, that is, leaving the hospital premises unexpectedly, without the knowledge of clinical staff, has been associated with poor clinical, social, and economic outcomes [2,3], putting both the patient and community at risk [4] and prolonging the recovery process [1]. Several reasons have been called into question to explain such a phenomenon, including feeling frightened by the inpatient unit or other patients [5,6,7], experiencing feelings of isolation or boredom during the hospital stay [8], and feeling worried about domestic problems and homesick for loved ones [6]. A recent systematic review pinpointed the desire for freedom from the psychophysical restrictions of the hospital as a potential reason for absconding [9], fueling the ongoing debate about the importance of guaranteeing greater freedom to patients to obtain greater treatment adherence [10]. In fact, coercive practices such as physical restraint and confinement are widely used in psychiatry, and there is concern about their iatrogenic potential [11].

‘Open-door policy’ refers to a policy of maintaining open doors in mental health settings and particularly hospital-based settings that otherwise would be ‘closed’ or ‘locked’ [12]. An improved therapeutic atmosphere and relationships with health-care staff have been suggested as potential advantages of open-door settings, allowing for less-restrictive but still secure treatment of patients at risk [13]. In this regard, longitudinal evidence does not support better performance of locked wards in preventing suicide and absconding [14].

When comparing dysfunctional behavior and containment methods among inpatient psychiatric units across Europe, the absconding rates are found to be the lowest in Italy [15]. Independently of cultural, social, and legislative reasons potentially explaining such difference, whether the reduced absconding rate in Italy reflects a less critical presentation in terms of aggressive behavior against others as well as the adoption of restraint measures remains unclear [15]. Therefore, the risk of absconding in open-door, no-restraint inpatient psychiatric units, remains to be tested. Additionally, understanding which patient categories are most at risk of absconding may help in anticipating the event and developing strategies to mitigate such risk. The aim of this retrospective study was twofold: (i) to estimate the rate of absconding from an open-door, no-restraint inpatient psychiatric unit in Italy; and (ii) to identify socio-demographic and clinical characteristics predicting an increased risk of absconding.

## 2. Materials and Methods

### 2.1. Care Setting

This study was conducted at the General Hospital Psychiatry Unit (GHPU), Mental Health Department, of the University Hospital of Udine. The GHPU responds to mental health emergencies requiring admission across an area of over 500,000 people. The GHPU adopted a non-restrictive policy in 2006, eliminating any mechanical restraints, and implemented an open-door policy in 2015, keeping the ward’s access door open thereafter. To avoid mechanical restraint utilization, healthcare providers adopt the following strategies: (i) to conduct a careful medical assessment of all patients at risk for acute agitation; (ii) whenever possible, to begin agitation management with verbal de-escalation techniques; and (iii) to use pharmacological therapies to help calm the patient and strengthen the effects of verbal de-escalation (e.g., benzodiazepines, antipsychotics).

### 2.2. Procedures

A retrospective analysis was conducted on the absconding from GHPU. All admissions in 2018–2021 were included. An observation with absconding was defined as voluntarily leaving the GHPU, in disagreement with health professionals, never to return, which led to the patient being discharged.

### 2.3. Collected Measures

For each hospitalization, the following information was recorded: (i) year, (ii) duration, (iii) patient’s age, (iv) sex, (v) ethnicity, (vi) whether at first contact with the GHPU, (vii) whether compulsorily admitted, and (viii) previous and (ix) current absconding. If present, services providing care for the patient were registered. Patients’ diagnoses were also recorded according to the International Statistical Classification of Diseases.

### 2.4. Data Analysis

The main unit of observation was the single hospitalization. In univariate analyses, Fisher’s, χ2-, *t*-, or the Mann–Whitney test were used, as appropriate. A Poisson’s regression model was then fitted on absconding from GHPU, introducing as covariates the measures that differentiated groups. Robust standard errors (SEs) and confidence intervals (CIs) were estimated. Incident rate ratios (IRRs) were calculated with the ∆-method. The model’s pseudo-R2, goodness of fit, and comparison with the corresponding negative binomial model were also reported. Analyses were conducted using R-4.2.0 (https://www.R-project.org accessed on 2 January 2023).

## 3. Results

Over the study period (2018–2021), a total of 949 patients were hospitalized in the GHPU (males: 50.1%; age at first hospitalization: 44.2 ± 15.56 years; non-Caucasian: 9.0%; already hospitalized before 2018: 28.5%). Additionally, 231 participants had multiple (2–16) hospitalizations, with a cumulative length of stay (LoS) of 13.2 ± 23.88 days per patient. The number of patients who absconded at least once in 2018–2021 was 43 (4.5%). They were more often male (81.4% vs. 48.6%; *p* < 0.001), younger (34.9 ± 11.82 vs. 44.6 ± 15.59 years-old; *p* < 0.001), and non-Caucasian (34.9% vs. 7.7%; *p* < 0.001). They also had more hospitalizations (2.8 ± 2.03 vs. 1.4 ± 1.13; *p* < 0.001) and a longer LoS (23.5 ± 40.35 vs. 12.7 ± 22.73; *p* = 0.010). Their admissions were most often for substance abuse (23.3% vs. 7.0%; *p* = 0.001), intellectual disability (14.0% vs. 4.6%; *p* = 0.018), and other/unclassified condition (11.6% vs. 3.8%; *p* = 0.027). No suicide was reported among absconding patients.

Table 1 summarizes hospitalization characteristics. Considering the differences between hospitalization groups, eleven measures were chosen as covariates (tolerances ranged from 0.576 to 0.938).

The model showed a reasonably good fit (χ21,390 = 247.28, *p* > 0.999; pseudo-R2 = 0.367) without the need to account for data dispersion (χ21 = −0.021, *p* > 0.999). The relative risk of absconding from the GHPU increased at a statistically significant level with: having already absconded (IRR: +4.342, 95% CI based on robust SE: [+2.38, +7.94]); being male (+4.227 [+2.03, +8.81]); being unknown to health services (+3.309 [+1.65, +6.65]); being compulsorily admitted (+2.874 [+1.53, +5.39]); being of non-Caucasian ethnicity (+2.661 [+1.56, +4.54]); being admitted for substance abuse (+2.480 [+1.27, +4.84]); being in the first days of hospitalization (+0.913 [+0.86, +0.97]); and being younger (+0.974 [+0.96, +0.99]). Fitted model details can be found in the Supplementary Material.

## 4. Discussion

This retrospective study investigated absconding rates and risk factors among patients admitted to an inpatient psychiatric unit adopting an open-door, no-restraint policy. Overall, the absconding rate was 4.5%, corroborating previous evidence that, as compared to coercive settings, absconding rates are not increased among less coercive settings and may actually be reduced [14,16]. In line with previous evidence, younger and male patients presented with a higher risk of absconding [8,16,17,18,19]. Additionally, non-Caucasian patients appeared to be at a greater risk of absconding, extending previous evidence regarding the excess mental health risk and poor health outcome among ethnic minorities, likely due to increased psychobiological disempowerment [20].

Moreover, as already indicated in previous reports [21,22,23], patients admitted because of substance abuse were more likely to abscond, calling into question abstinence issues [22]. Further, it is not surprising that patients compulsorily admitted had a higher risk of absconding, potentially reflecting poor treatment adherence as well as a more severe clinical presentation [24].

Despite expectations [19], the finding that those who had already experienced absconding from the inpatient unit were more likely to abscond again raises the question of implementing strategies to mitigate, if not prevent, such risk. Independent of other potential options, the current study offers itself a way of tackling the risk of multiple absconding. In fact, the risk of absconding was found to be significantly higher in patients unknown to community services, suggesting that the latter may represent a suitable network to sustain adherence to inpatient stay. Additionally, as most patients were known to services and we did not observe any suicide, further studies will have to explore the role of community services in avoiding fatal events among absconding patients. This seems to be of paramount importance as coercive settings have been suggested not to be able to prevent suicide [14].

The limitations of this study include the absence of data from coercive settings or from the same inpatient unit prior to the implementation of the open-door, no-restraint policy. The latter in particular would have offered a comparison of trends in absconding as a function of the policy change. Additionally, the investigation was carried out in a single hospital, limiting the generalizability of the findings. Furthermore, we cannot exclude that those patients going absent without leaving but found in the hospital premises and returned to the inpatient unit may not have been recorded as absconding patients, underestimating the absconding rates. Finally, apart from recording the disorder for which the patient was admitted to the ward, information on the clinical severity was not collected, nor was the reason for absconding, requiring investigation in further studies. Nevertheless, the findings of this study may have important public health implications as they suggest that specific patient populations may be at higher risk of absconding, even in an open-door, no-restraint psychiatric unit presenting with relatively low absconding rates.

## Figures and Tables

**Table 1:** Hospitalization characteristics over the study period (2018–2021).

	Without Absconding	With Absconding	Comparison
Hospitalization:			
Number (%)	1339 (95.5%)	63 (4.5%)	-
Year: 2018	377 (28.2%)	16 (25.4%)	OR = 0.87 [0.45–1.58], *p* = 0.774
2019	332 (24.8%)	20 (31.7%)	OR = 1.41 [0.77–2.49], *p* = 0.234
2020	295 (22.0%)	11 (17.5%)	OR = 0.75 [0.35–1.48], *p* = 0.439
2021	335 (25.0%)	16 (25.4%)	OR = 1.02 [0.53–1.86], *p* > 0.999
Duration in days	9.1 ± 15.66 (0.2–299.0)	4.7 ± 5.90 (0.3–25.0)	t_109.4_ = +5.16, *p* < 0.001 ***
Compulsory hospitalization	147 (11.0%)	15 (23.8%)	OR = 2.53 [1.28–4.74], *p* = 0.004 **
Hospitalized patients:			
Sex (male)	643 (48.0%)	55 (87.3%)	OR = 7.43 [3.48–18.21], *p* < 0.001 ***
Age in years	43.4 ± 15.15 (11–86)	33.4 ± 11.13 (18–61)	U = 58283.5, *p* < 0.001 ***
Age-group (years-old): <30	304 (22.7%)	30 (47.6%)	OR = 3.09 [1.79–5.32], *p* < 0.001 ***
30–40	255 (19.0%)	17 (27.0%)	OR = 1.57 [0.83–2.85], *p* = 0.141
41–50	337 (25.2%)	12 (19.0%)	OR = 0.70 [0.34–1.35], *p* = 0.300
>50	443 (33.1%)	4 (6.3%)	OR = 0.14 [0.04–0.37], *p* < 0.001 ***
Non-Caucasian ethnicity	108 (8.1%)	24 (38.1%)	OR = 7.00 [3.87–12.44], *p* < 0.001 ***
Already known to the GHPU	682 (50.9%)	41 (65.1%)	OR = 1.80 [1.03–3.20], *p* = 0.029 *
Other episodes of absconding	34 (2.5%)	20 (31.7%)	OR = 17.74 [8.92–34.79], *p* < 0.001 ***
Sending Service:			
Community Mental Health Centres	1147 (85.7%)	49 (77.8%)	OR = 0.59 [0.31–1.17], *p* = 0.099
Drug Addiction Services	73 (5.5%)	7 (11.1%)	OR = 2.17 [0.80–4.99], *p* = 0.085
Private	53 (4.0%)	0 (0.0%)	OR < 0.01 [<0.01–1.52], *p* = 0.168
Disability Services	7 (0.5%)	0 (0.0%)	OR < 0.01 [<0.01–14.99], *p* = 1.000
Child/Adolescent Mental Health Centres	6 (0.4%)	0 (0.0%)	OR < 0.01 [<0.01–18.33], *p* = 1.000
Unknown	77 (5.8%)	9 (14.3%)	OR = 2.73 [1.14–5.85], *p* = 0.012 *
Reason for hospitalization:			
Non-affective psychotic disorder	407 (30.4%)	20 (31.7%)	OR = 1.07 [0.59–1.88], *p* = 0.889
Mood/Affective disorder	359 (26.8%)	11 (17.5%)	OR = 0.58 [0.27–1.14], *p* = 0.109
Anxiety and somatoform disorder	280 (20.9%)	6 (9.5%)	OR = 0.40 [0.14–0.93], *p* = 0.025 *
Personality disorder	95 (7.1%)	5 (7.9%)	OR = 1.13 [0.35–2.89], *p* = 0.800
Substance abuse disorder	81 (6.0%)	10 (15.9%)	OR = 2.93 [1.28–6.09], *p* = 0.006 **
Intellectual disability	59 (4.4%)	6 (9.5%)	OR = 2.28 [0.77–5.58], *p* = 0.067
Physiological condition	20 (1.5%)	0 (0.0%)	OR < 0.01 [<0.01–4.36], *p* = 1.000
Other/Unclassified condition	38 (2.8%)	5 (7.9%)	OR = 2.95 [0.87–7.90], *p* = 0.040 *

Number of observations or mean and standard deviation are reported (with percentages or 95% Confidence interval, CI, between brackets); statistical significance of univariate comparisons is also reported; CI, confidence interval; GHPU, General Hospital Psychiatry Unit; OR, odds ratio (with 95% CI between square brackets); statistically significant with *, *p* < 0.050; **, *p* < 0.010; ***, *p* < 0.001.

## Data Availability

The data presented in this study are available on request from the corresponding author.

## Supplementary Materials

The following supporting information can be downloaded at: https://www.mdpi.com/article/10.3390/bs13010058/s1, Table S1: Multivariate analysis: Poisson’s regression on absconding from GHPU.
