# Supplementary material for: Prevalence and Risk Factors for Absconding from an Open-Door, No-Restraint Inpatient Psychiatric Unit: A Single-Center Study in Italy
Source: Behav Sci (Basel). 2023 Jan 8;13(1):58. doi: 10.3390/bs13010058 (PMC9854979; doi:10.3390/bs13010058)
Supplement: Supplementary file 1 [file behavsci-13-00058-s001.zip › behavsci-2091366-supplementary.pdf]

Supplementary Table S1. Multi-variate analysis: Poisson's regression on absconding from GHPU.

Predictor	Coefficient ±SE	±95% CI		IRR ±SE	±95% CI
Intercept	-3.490 ±0.521	[-4.512, -2.468]	***	+0.031 ±0.016	[+0.011, +0.085]
Duration of hospitalization (days)	-0.091 ±0.031	[-0.152, -0.030]	**	+0.913 ±0.028	[+0.859, +0.971]
Compulsory hospitalization (=1)	+1.056 ±0.321	[+0.427, +1.684]	***	+2.874 ±0.921	[+1.533, +5.387]
Male (=1)	+1.442 ±0.375	[+0.708, +2.176]	***	+4.227 ±1.583	[+2.029, +8.808]
Age at hospitalization (years)	-0.026 ±0.009	[-0.044, -0.008]	**	+0.974 ±0.009	[+0.957, +0.992]
Non-Caucasian ethnicity (=1)	+0.979 ±0.272	[+0.445, +1.512]	***	+2.661 ±0.724	[+1.561, +4.537]
Already known to the PU (=1)	+0.274 ±0.296	[-0.306, +0.855]		+1.315 ±0.390	[+0.736, +2.351]
Other episodes of absconding (=1)	+1.468 ±0.308	[+0.865, +2.072]	***	+4.342 ±1.337	[+2.375, +7.940]
Unknown to health services (=1)	+1.197 ±0.356	[+0.498, +1.895]	***	+3.309 ±1.179	[+1.646, +6.651]
Anxiety and somatoform disorder (=1)	-0.616 ±0.455	[-1.508, +0.276]		+0.540 ±0.246	[+0.221, +1.318]
Substance abuse (=1)	+0.908 ±0.341	[+0.240, +1.577]	**	+2.480 ±0.846	[+1.271, +4.840]
Other/Unclassified condition (=1)	+0.548 ±0.407	[-0.250, +1.347]		+1.730 ±0.705	[+0.779, +3.846]

CI, Confidence Interval (calculated using robust standard errors); GHPU, General Hospital Psychiatry Unit; IRR, Incident Rate Ratios (calculated with Δ-method); SE, Robust Standard Error; Statistical significance of the predictors, with: *, p<0.050; **, p<0.010; ***, p<0.001
